# Association between Renal Podocalyxin Expression and Renal Dysfunction in Patients with Diabetic Nephropathy: A Single-Center, Retrospective Case-Control Study

**DOI:** 10.1155/2020/7350781

**Published:** 2020-04-03

**Authors:** Rongzhen Wang, Can Yao, Feng Liu

**Affiliations:** ^1^Department of Nephropathy, The First Hospital of Lanzhou University, Lanzhou, Gansu Province, China; ^2^Institute of Pathology, Basic Medical College of Lanzhou University, Lanzhou, Gansu Province, China

## Abstract

This retrospective study investigated whether podocalyxin expression in renal biopsies and urine of patients with diabetic nephropathy (DN) is associated with renal function. This retrospective study included 32 patients with nephropathy, secondary to type 2 diabetes treated at the First Hospital of Lanzhou University (January 2010 to January 2015). Compared with the control group, the DN group had a significantly lower renal expression of podocalyxin and higher urinary podocalyxin/creatinine ratio. Patients with DN were divided into the high and low expression groups according to podocalyxin expression in renal tissues. Patients in the low expression group had longer diabetes duration, lower plasma albumin and eGFR, higher glycated hemoglobin (HbA1c), 24 h urinary protein, serum creatinine, and urinary podocalyxin/creatinine ratio, and more severe glomerular, tubulointerstitial, and renal interstitial inflammation than patients in the high expression group (all *P* < 0.01). The renal survival rate was significantly lower in the low expression group than in the high expression group (*P* < 0.01). Single-factor Cox regression analysis showed that reduced podocalyxin expression and increased urinary podocalyxin excretion were associated with poor renal outcome. Measuring podocalyxin levels in renal tissues and urine could help evaluate the progression of DN.

## 1. Introduction

Diabetes mellitus (DM) is a metabolic disease associated with hyperglycemia due to abnormalities of insulin secretion, action, or both [1]. The prevalence of type 2 DM (T2DM) has increased in recent years because of population aging and lifestyle changes [2]. Globally, around 382 million patients were living with DM in 2013, and it has been predicted that this will increase to nearly 600 million by 2035 [3]. DM is common in China, where the age-standardized prevalence of diabetes/prediabetes is 9.7%-11.6% [4, 5]. Diabetic nephropathy (DN) is now the most common cause of end-stage renal disease (ESRD) in China [6], the USA [7], and Europe [8]. DN develops in up to 40% of patients with type 1 DM [9] and 25% of individuals with T2DM [10].

DN is the renal manifestation of systemic microangiopathy and one of the major causes of mortality and disability in patients with T2DM. DN is characterized by a gradual increase in the urinary albumin excretion rate (UARE), and kidney function progressively worsens until ESRD develops. Previous studies suggested that alterations in the components of the glomerular basement membrane (GBM) and aggregation of the extracellular matrix (ECM) are key changes that occur in DN [11, 12]. However, these typical changes cannot completely explain the occurrence of an abnormal UARE in DN.

Studies in recent years have shown that damage to podocytes plays an essential role in the pathogenesis of DN [13]. Podocytes are a type of ultimately differentiated visceral epithelial cell located on the lateral side of the glomerular capillaries, and they form the primary structure of the glomerular filtration barrier. The slit diaphragm is the last barrier preventing the loss of protein from the glomerular filtrate. Since injury to the podocytes or slit diaphragms contributes to the development of proteinuria and DN, the identification of a reliable indicator reflecting damage to podocytes or slit diaphragm could potentially allow the prediction of the progression of DN.

Podocalyxin (PCX) is a podocyte membrane protein and the major negatively charged protein in the glomeruli. PCX is the main component of the charge barrier of the glomerular basement membrane (GBM) and plays a critical role in regulating the permeability of the glomerular filtration barrier [14]. Previous studies have shown that urinary PCX is associated with podocyte damage in patients with DN and could be used as an early indicator for the diagnosis of DN [15]. Nevertheless, very few studies investigated the associations of PCX expression in renal tissues and PCX levels in urine with the progression of renal dysfunction in patients with DN. Therefore, this study is aimed at investigating the associations of PCX expression in renal tissues and PCX levels in urine with the progression of proteinuria and renal dysfunction in patients with DN and exploring whether renal or urinary PCX might be a reliable marker for predicting the progression of DN.

## 2. Patients and Methods

### 2.1. Study Design and Patients

This retrospective analysis included 32 patients with T2DM and DN treated at the First Hospital of Lanzhou University between January 2010 and January 2015. The protocol for this retrospective case-control study was approved by the ethics committee of our hospital (LDYYLL2019-217). This work has been carried out in accordance with the Declaration of Helsinki (2000) of the World Medical Association. All patients provided informed written consent. T2DM and DN were confirmed by clinical and pathological diagnoses [1, 16], and the estimated glomerular filtration rate (eGFR) of all patients was >60 mL/min/1.73 m^2^. DN was diagnosed based on (1) persistent albuminuria confirmed at least two times 3-5 months apart, (2) a decline in eGFR, (3) elevated blood pressure, and (4) exclusion of other causes of albuminuria [16]. The indication for biopsy is eGFR > 60 mL/min/1.73 m^2^ [16]; all patients included here underwent renal biopsy. The exclusion criteria were (1) primary or other secondary renal diseases and (2) lost to follow-up. All participants provided informed written consent. In addition to the above participants with T2DM, six control specimens of healthy renal tissue were obtained from patients with renal tumors who had been treated surgically at the Urology Department, and urine samples were obtained from 12 subjects attending the Outpatient Department for health examinations. All 12 subjects who provided control urine samples were healthy and without hypertension or T2DM, and all had a normal renal function, no urine sample abnormalities, and normal levels of glycated hemoglobin (HbA1c).

### 2.2. Collection of Clinical Data

Data including age at renal biopsy, sex, diabetes disease course, nephropathy disease course, blood pressure, HbA1c, fasting blood glucose (FPG), cholesterol, triglyceride, serum creatinine (SCr), plasma albumin (ALB), and urinary N-acetyl-beta-D-glucosaminidase (NAG) were collected. eGFR was calculated according to the Chronic Kidney Disease-Epidemiology Collaboration (CKD-EPI) equation [17]. A 24 h urine sample was collected on the day before the renal biopsy, and 24 h urinary protein quantity was measured. Urine samples (24 h and morning) were collected, centrifuged, and stored at -20°C. They were tested in batches, all at the same time. All patients with T2DM were followed in our hospital after discharge, and SCr and eGFR were recorded at the last follow-up.

### 2.3. Pathological Data

The renal tissues were examined by light microscopy, immunofluorescence (IF) microscopy after frozen sectioning, and electron microscopy. The glomeruli, tubulointerstitium, and blood vessels were graded or scored according to [18, 19]. All pathological pictures were evaluated by two experienced kidney pathologists.

### 2.4. Measurement of PCX in Renal Tissues

The expression of PCX in renal tissues was measured using indirect immunofluorescence. The specimens obtained by renal biopsy were processed to frozen sections with a thickness of 5 *μ*m, dried at room temperature for 30 min, fixed by acetone at 4°C for 10 min, and rinsed with phosphate-buffered saline (PBS) three times. The sections were blocked with serum from unrelated animals at room temperature for 20 min, the serum was then discarded, and the unlabeled primary antibody (Santa Cruz Biotechnology Inc., sc-23904) was added. After incubation at 37°C for 30 min, the sections were rinsed with PBS three times, and fluorescein-labeled secondary antibody (fluorescein-labeled goat anti-mouse IgG; 1 : 100, ZSGB-BIO, SP-9001) was added. The sections were incubated at 37°C for another 30 min and then rinsed with PBS three times. The intensity of the fluorescence in the glomeruli was observed by fluorescence microscopy. The fluorescence intensity was classified as highly positive (+++) for extremely high intensity, positive (++) for high intensity, weakly positive (+) for some fluorescence, and negative (–) for no fluorescence. All images were analyzed by two renal pathologists who were of attending physician grade or higher.

### 2.5. Quantification of Urinary PCX

PCX in the urine was measured using an enzyme-linked immunosorbent assay (ELISA) kit (MyBioSource, USA), in accordance with the instructions of the manufacturer. In order to adjust for the influence of urine volume, the results were expressed as the ratio of urinary PCX to that of urinary creatinine (PCX/UCr ratio, *μ*g/mg).

### 2.6. Definitions

The diabetes course was defined as the time from the diagnosis of T2DM to renal biopsy (months). The nephropathy disease course was defined as the time from the diagnosis of renal disease to renal biopsy (months). The endpoint events included eGFR < 15 mL/min/1.73 m^2^, hemodialysis for over three months, or eGFR reduced by >50% accompanied by eGFR > 60 mL/min/1.73m^2^.

### 2.7. Statistical Analysis

SPSS 24.0 software (IBM Corp., USA) was used for data analysis. Quantitative data were tested for normality. Quantitative data with a normal distribution are described as the mean ± standard deviation and were compared between groups using Student's *t*-test. Quantitative data with a nonnormal distribution are presented as median (quartile) and were compared between groups using the Wilcoxon rank-sum test. Qualitative data are described as percentages and were compared between groups using Fisher's exact test. Spearman correlation or Pearson correlation tests were used to assess associations between parameters. The Kaplan-Meier method and log-rank test were used for renal survival analysis. A univariable Cox regression model was used to identify factors associated with renal outcomes. *P* < 0.05 was considered statistically significant.

## 3. Results

### 3.1. PCX Expression in Renal Tissues and Urine

**Figure 1:**
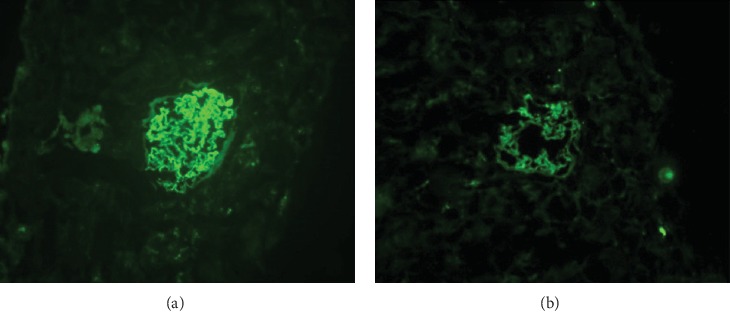
Detection of podocalyxin in renal tissue using immunofluorescence methods. (a) Intense expression of podocalyxin (granular or linear pattern) in normal glomerular capillary loops. (b) In damaged glomeruli, podocalyxin immunofluorescence was reduced in intensity and uneven in distribution, with regions of deficient expression.

The expression of PCX in renal tissues was measured by IF (Figure 1). In control specimens, PCX was distributed evenly along the glomerular capillary walls, and the fluorescence intensity was highly positive (+++) (Figure 1(a)). Although PCX was also expressed in the renal tissues from all 32 patients with DN, its distribution in the glomeruli of patients with DN was uneven and discontinuous with a segmental deficiency in some areas, and the fluorescence intensity was lower than that of controls (Figure 1(b)).

ELISA detected PCX in urine samples from patients with DN but not in the control urine samples. Urinary PCX/UCr was significantly higher for patients with DN than for control subjects (53.19 ± 14.29 vs. 0 *μ*g/mg; *P* < 0.01).

### 3.2. Subgroup Analysis Based on the Level of PCX Expression in Renal Tissues from Patients with DN

We further categorized the patients with DN into a low expression group (intensity of +; *n* = 14) and a high expression group (intensity of ++ or +++; *n* = 18). Patient age, patient sex, nephropathy disease course, mean arterial pressure, and the levels of NAG, FPG, cholesterol, and triglycerides were not significantly different between the two groups. Patients in the low expression group had significantly longer diabetes disease course, higher HbA1c levels, lower plasma ALB levels, lower eGFR, higher 24 h urinary protein quantity, higher SCr, and higher urinary PCR/UCr ratio than patients in the high expression group (all *P* < 0.01; Table 1). In addition, the levels of inflammation in the glomeruli, tubulointerstitium, and renal interstitium were all significantly higher in the low expression group than in the high expression group (all *P* < 0.01). The percentage of patients with arteriosclerosis was also higher in the low expression group than in the high expression group (*P* < 0.01; Table 2).

### 3.3. Associations between PCX Expression and Clinicopathological Characteristics

Spearman correlation analysis was used to assess the associations between renal PCX expression and clinicopathological characteristics, and Pearson correlation analysis was used to assess the associations between urinary PCX/UCr ratio and clinicopathological characteristics (Table 3). Renal PCX expression level was negatively correlated with urinary PCX/UCr ratio (rs = −0.695, *P* < 0.001). Furthermore, lower renal PCX expression (rs = −0.706, *P* < 0.001) and higher urinary PCX/UCr ratio (*r* = 0.534, *P* = 0.002) were correlated with increased HbA1c. Decreased PCX expression was also associated with higher urinary protein levels, lower plasma ALB, decreased eGFR, and more severe tubule interstitial inflammation and arteriosclerosis (*P* < 0.01; Table 3). There were no significant correlations of renal PCX expression or urinary PCX/UCr ratio with age, mean arterial pressure, blood glucose level, or blood lipid level.

### 3.4. Association between PCX Expression and Renal Outcome

**Figure 2:**
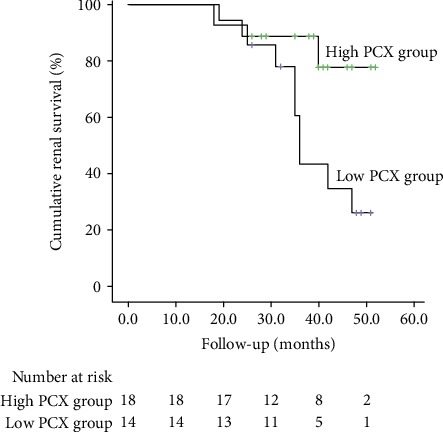
Kaplan-Meier renal survival curves for patients in the low expression group and high expression group. Cumulative renal survival (%); follow-up time (months); high expression group; low expression group.

The patients with DN in the high expression group were followed for 53.2 ± 13.4 months, and those in the low expression group were followed for 52.7 ± 15.4 months. Kaplan-Meier analysis showed that the renal survival rate was significantly higher in the high expression group than in the low expression group (*P* = 0.032; Figure 2). Univariate Cox regression analysis showed that lower plasma ALB, higher NAG, higher SCr, higher 24-hour urinary protein quantity, lower eGFR, lower renal PCX expression, and higher urinary PCX/UCr ratio were factors associated with poor renal outcome in the 32 patients with DN (Table 4).

## 4. Discussion

An important finding of the present study was that patients with DN had lower renal expression of PCX and higher urinary PCX/UCr ratio than healthy subjects. Furthermore, patients with DN in the low PCX expression group had a longer diabetes disease course, lower plasma ALB and eGFR, higher HbA1c, 24 h urinary protein, SCr, and urinary PCX/UCr ratio, and more severe glomerular, tubulointerstitial, and renal interstitial inflammation than patients in the high expression group. The renal survival rate was significantly lower in the low expression group than in the high expression group. Notably, univariable Cox regression analysis showed that reduced renal PCX expression and increased urinary PCX excretion were associated with poor renal outcome. Taken together, our findings suggest that the progression of DN could potentially be evaluated from the measurements of PCX levels in renal tissues and urine. Nevertheless, based on the data of the present study, it cannot be ascertained, without any doubt, that the differences observed between the two groups are due to PCX underexpression and not due to generalized diabetic podocytopathy. This will have to be further studied.

DN is a common but severe complication of diabetes and accounts for about 40%-50% of all cases of ESRD in developed countries. It is estimated that about 500 million patients will live with T2DM in 2030, one-third to one-quarter of them will progress to DN, and about 20% of those with DN will progress to ESRD [20]. Proteinuria is closely associated with the prognosis of patients with DN. Recent studies concluded that podocyte damage plays a vital role in the pathogenesis of DN [13, 21]. Podocytes are located in the outer layer of the GBM and help maintain the integrity of the glomerular filtration barrier. PCX is the major negatively charged protein in glomeruli and the major component of the charge barrier of the GBM. PCX is synthesized in glomerular endothelial cells and podocytes and is restricted to the apical membrane during the maturation of podocytes. The charges on the surface of PCX have antiadhesive effects, and PCX is anchored to the apical membrane by structural proteins such as cytoskeleton actin. Maintenance of the structures of the podocytes and slit diaphragms is essential to prevent negatively charged proteins from entering the urine through the slit diaphragms.

Glycated products are involved in the pathogenesis of T2DM, and HbA1c levels are a recognized marker of glycemic control in patients with T2DM [22, 23]. On the other hand, fasting blood glucose levels are more variable and easily affected by the length of fast, activity level, and various drugs. The measurement of blood glucose is often necessary to avoid hypoglycemia or hyperglycemia events, but it does not represent long-term glycemic control, as HbA1c does [22, 23]. Petrica et al. [24] indicated that glycated products could affect the podocytes and proximal tubules and lead to an increase in urinary PCX levels. In the present study, HbA1c levels were associated with low expression of PCX, and HbA1c levels were inversely correlated with urinary PCX levels, as supported by previous studies [24–26].

PCX plays a critical role in regulating the permeability of the glomerular filtration barrier. Chemical modification of PCX can damage the filtration barrier and have direct effects on the stability of podocyte processes. In specific diseases, podocytes are also targeted during inflammatory and noninflammatory injury to the glomeruli. Damaged podocytes exhibit morphological changes and can detach into the urine. The exposed basement membrane then contacts with the parietal epithelial cells of the glomerular capsule, which leads to capsular synechia and glomerular sclerosis.

In this study, the PCX expression level in renal tissues was significantly lower for patients with DN than for control subjects without DN. Patients with DN were further divided into high and low expression groups according to the PCX expression level. Importantly, ALB, 24 h urinary protein quantity, SCr, and eGFR were significantly different between the two groups. The above results suggest that decreased renal expression of PCX may be an indicator of podocyte damage, which in turn could lead to glomerulopathy, disruption of the GBM, elevated urinary levels of protein, increased severity of tubulointerstitial fibrosis, and interstitial inflammation, and finally, a decrease in renal function. Podocyte damage is a risk factor for poor renal outcomes. Previous studies have shown that hyperglycemia, genetic background, proteinuria, chronic oxidative stress-inflammatory state, and various complications are factors associated with podocyte damage [27–29]. PCX on the surface of podocytes is a CD34-related sialomucin that contains large amounts of sialic acid and sulfate. The structure of PCX contains a mucoprotein domain, a spherical domain formed by a disulfide bond, a transmembrane domain, and an intracellular domain with a large amount of charge. The intracellular domain contains potential phosphorylation sites for PKC and tyrosine kinase II. Cortactincan interact with the actin filaments of podocytes through the regulation of PCX. Nevertheless, the factors that can induce damage to PCX remain unclear. Our findings showed that a reduction in PCX expression was significantly associated with increased urinary protein, reduced eGFR, and increased HbA1c. Hence, these factors may contribute to podocyte damage. The expressions of mRNA and protein PCX in podocytes cultured in high-glucose medium are both suppressed. The mechanisms underlying podocyte damage are complex, and various factors such as hyperglycemia, angiotensin II, transforming growth factor-*β*, mechanical stress, mitotic mutation, and oxidative stress play important roles [28, 30–34]. PCX plays a crucial role in the formation and maintenance of the cytoskeleton of podocytes through regulation of the NHER F2/ezrin complex. Therefore, a reduction in PCX expression disrupts the interaction with the actin cytoskeleton, which consequently induces pathological changes in podocytes that promotes the development and progression of DN.

Since DN represents a continuum of lesions of variable severity among patients, and since the aggressiveness of treatments should be determined based on this severity, the adequate and precise assessment of the extent of kidney injury in patients with DN is important for optimal patient management [20, 35]. Hence, the levels of PCX in kidney tissues and urine could be used to tailor the treatment of DN to each specific patient. Indeed, Petrica et al. [24] showed that increased PCX levels were associated with proximal tubule dysfunction independently from albuminuria and eGFR decline. Ye et al. [36] and Hara et al. [37] showed that positive urinary PCX could be used as a noninvasive marker of early-stage DN. The urinary and plasma levels of some miRNAs were associated with the urinary levels of PCX and were also associated with proximal tubule injury [38, 39]. Metformin has been shown to restore PCX expression in diabetic rats [40], but a study suggested that dipeptidyl peptidase-4 (DDP-4) inhibitors and *α*-glucosidase inhibitors did not change the urinary levels of PCX [41]. The impact of available antidiabetic, antihypertensive, and renal protective treatments on PCX expression and urinary levels will have to be determined in relation to prognosis.

A follow-up of the 32 patients in this study showed that the renal survival rate was significantly lower in patients with lower renal PCX expression than in patients with higher renal PCX expression. Further analysis revealed that PCX expression in renal tissues was a factor associated with the progression of renal function. Therefore, measuring the expression levels of PC in the kidneys could potentially predict the renal outcome. ALB, NAG, SCr, 24 h urinary protein quantity, and eGFR were also factors associated with the progression of renal dysfunction. Furthermore, tubular atrophy, renal interstitial fibrosis, and renal inflammation score were associated with poorer renal function. Previous research has demonstrated that tubular atrophy and renal interstitial fibrosis participates in the progression of DN and are closely associated with the reduction in renal function [42].

Another finding of this study was that urinary excretion of PCX increased as the renal expression of PCX decreased, which would be consistent with the detachment of PCX protein from renal tissue into the urine. Previous investigations determined that an increased PCX level in the urine could be used as a biomarker to predict early renal damage as well as the development and progression of complications in patients with early DN, anaphylactic purpura nephritis, lupus nephritis, or IgA nephropathy [15, 43–47]. In addition, measuring PCX in urine is a noninvasive method that could be readily implemented in the clinical setting. Although previous studies focused on patients with early-stage DN, the present study included patients with DN and clinical albuminuria. The finding that urinary PCX levels were increased in our patients and significantly associated with a reduction in renal function suggests that the potential clinical utility of this parameter as a biomarker might not be limited to patients with early-stage DN.

This study has some limitations. First, this was a retrospective case-control study, so it may have been prone to selection bias and information bias. Second, this was a single-center study and, because the indications for renal biopsy are limited, the sample size was small, and the generalizability of our findings is unknown. Third, the small sample size precluded a multivariable analysis and may have resulted in our analyses being underpowered to detect some real differences between groups. Large-scale prospective studies are needed to validate and extend our results. Because of the limited medical conditions in China, many patients are diagnosed late in the course of their disease, leading to the discrepancy between the diagnosis and the actual start of the disease. Hence, the results of the courses of T2DM and DN have to be taken with caution. Finally, only patients with DN were included, and future studies should examine PCX in the full continuum of kidney lesions found in patients with T2DM, including patients with some extent of kidney damage but without albuminuria.

## 5. Conclusion

The findings of this study raise the possibility that PCX levels in renal tissues and urine could be used to predict the progression of renal dysfunction and renal outcomes in patients with DN.

## Figures and Tables

**Table 1:** Baseline clinical characteristics of the patients with diabetic nephropathy at the time of renal biopsy.

	High expression group (*n* = 18)	Low expression group (*n* = 14)	*P* value
Age (years)	53.9 ± 5.6	56.0 ± 8.4	0.401
Male/female	9/9	8/6	0.735
Course of diabetes (months)	50.5 (30.5-72.8)	78 (47.7-95.5)	0.035
Course or nephropathy (months)	24.9 ± 18.5	30.5 ± 17.9	0.395
Mean arterial pressure (mmHg)	104 ± 13.6	107.9 ± 16.1	0.454
HbA1c (%)	6.98 ± 0.86	8.25 ± 0.58	<0.001
FPG (mmol/L)	7.80 ± 1.34	8.15 ± 1.24	0.456
Triglycerides (mmol/L)	2.76 ± 1.05	2.91 ± 0.93	0.66
Cholesterol (mmol/L)	5.40 ± 0.92	5.44 ± 0.91	0.914
Albumin (g/L)	35.7 ± 5.0	30.0 ± 3.7	0.001
Urinary NAG (U/L)	32.4 ± 8.7	34.9 ± 9.5	0.462
Urinary protein (g/24 h)	2.32 ± 1.00	3.38 ± 0.99	0.006
Serum creatinine (*μ*mol/L)	68.7 ± 10.3	89.3 ± 16.2	<0.001
eGFR	94.8 ± 16.2	74.1 ± 13.5	0.001
Urinary PCX/UCr	45.3 ± 11.1	63.2 ± 11.5	<0.001
ACE-i or ARB	18 (100.0)	14 (100.0)	>0.99

eGFR: estimated glomerular filtration rate; FPG: fasting plasma glucose; HbA1c: glycated hemoglobin; NAG: N-acetyl-beta-D-glucosaminidase; PCX: podocalyxin; UCr: urinary creatinine; ACE-i: angiotensin-converting enzyme inhibitor; ARB: angiotensin receptor blocker.

**Table 2:** Pathology findings for patients with diabetic nephropathy who underwent renal biopsy.

	High expression group (*n* = 18)	Low expression group (*n* = 14)	*P* value
Glomerular grade			<0.001
I	1	1	
II	7	6	
III	10	7	
IFTA			0.003
0	4	3	
1	6	4	
2	5	4	
3	3	3	
Renal interstitial inflammation			<0.001
0	8	1	
1	9	6	
2	1	7	
Arteriosclerosis			<0.001
0	2	0	
1	13	8	
2	3	6	

eGFR: estimated glomerular filtration rate; IFTA: tubular atrophy and interstitial fibrosis.

**Table 3:** Associations of podocalyxin expression in renal tissues and urinary podocalyxin/urine creatinine ratio with the clinicopathological characteristics of patients with diabetic nephropathy.

	Renal podocalyxin expression		Urinary podocalyxin/urinary creatinine	
	Correlation coefficient	*P* value	Correlation coefficient	*P* value
Age (years)	-0.127	0.489	0.222	0.221
Diabetes course (months)	-0.273	0.137	0.162	0.376
Nephropathy course (months)	-0.155	0.389	0.012	0.949
Mean arterial pressure (mmHg)	-0.151	0.408	0.072	0.649
HbA1c (%)	-0.706	<0.001	0.534	0.002
FPG (mmol/L)	-0.206	0.257	0.209	0.25
Triglycerides (mmol/L)	-0.203	0.264	0.304	0.091
Cholesterol (mmol/L)	-0.112	0.541	0.102	0.578
Albumin (g/L)	0.559	0.001	-0.629	<0.001
NAG (U/L)	-0.205	0.260	0.343	0.05
Urine protein (g/24 h)	-0.513	0.003	0.764	<0.001
Serum creatinine (*μ*mol/L)	-0.629	<0.001	0.614	<0.001
eGFR	0.604	<0.001	-0.677	<0.001
Glomerular grade	-0.108	0.556	0.268	0.138
IFTA	-0.325	0.069	0.49	0.004
Renal interstitial inflammation	-0.547	0.001	0.562	0.001
Renal arteriosclerosis	-0.386	0.029	0.441	0.011
Podocalyxin	-0.691	<0.001	-0.695	<0.001

eGFR: estimated glomerular filtration rate; FPG: fasting plasma glucose; HbA1c: glycated hemoglobin; IFTA: tubular atrophy and interstitial fibrosis; NAG: N-acetyl-beta-D-glucosaminidase.

**Table 4:** Univariate Cox regression analysis of factors associated with renal survival in patients with diabetic nephropathy.

	HR	95% Cl	*P* value
Albumin	0.86	0.757-0.976	0.020
NAG	1.093	1.022-1.169	0.010
Urinary podocalyxin/urinary creatinine	1.069	1.020-1.120	0.005
Serum creatinine	1.044	1.011-1.078	0.009
Renal podocalyxin grade	0.278	0.080-0.968	0.044
IFTA	2.583	1.186-5.625	0.017
Inflammation of renal interstitium	2.191	0.965-4.973	0.061
24 h urinary protein quantity	2.125	1.108-4.073	0.023
eGFR	0.939	0.898-0.982	0.006

95% CI: 95% confidence interval; eGFR: estimated glomerular filtration rate; HR: hazard ratio; IFTA: tubular atrophy and interstitial fibrosis; NAG: N-acetyl-beta-D-glucosaminidase.

## Data Availability

The data used to support the findings of this study are available from the corresponding author upon request.
